# Prone position effect in intensive care patients with SARS-COV-2 pneumonia

**DOI:** 10.1515/med-2023-0735

**Published:** 2023-06-27

**Authors:** Sandra Manuela Rebelo Oliveira, Alexandra Marisa da Silva Ferreira, Paulo Jorge Ventura Silva, Cristina Susana Sousa Pinto, Maria Glória Cabral Campello, Amâncio António de Sousa Carvalho

**Affiliations:** Tâmega and Sousa Hospital Center, Intensive Care Unit, Penafiel, Portugal; Department of Health School, Health School, University of Trás-os-Montes e Alto Douro, Vila Real, Portugal; CIEC - Research Centre on Child Studies, University of Minho, Braga, Portugal

**Keywords:** COVID-19, SARS-Cov-2, respiratory distress syndrome, prone position

## Abstract

Ventilation in the prone position (PP) has been used for decades in patients with acute respiratory distress syndrome (ARDS) and is associated with a reduction in mortality rate. Its application has been extended to patients with SARS-Cov-2 pneumonia and is recommended by the main international organizations. The objective is to evaluate the effects of PP on the outcomes of patients with SARS-Cov-2 pneumonia admitted to a multipurpose intensive care unit. This is a quantitative, quasi-experimental, single-group, longitudinal and retrospective study. Data were collected based on clinical records. Data were processed using SPSS (version 26.0). PP significantly increased oxygenation in patients with SARS-Cov-2 pneumonia, with a mean increase of 21.27% between the PaO_2_/FiO_2_ ratio before and after the PP. However, its effectiveness was inversely proportional to the number of cycles performed and the timing of orotracheal intubation. PP improves oxygenation in patients with SARS-Cov-2 pneumonia. However, multiple PP sessions are not beneficial, as this procedure is no longer effective after the fourth cycle. This study thus contributes to better management in the approach of critically ill patients with SARS-Cov-2 pneumonia.

## 1 Introduction

Severe acute respiratory syndrome coronavirus 2 (SARS-CoV-2), which emerged as a new human pathogen in China in late 2019, is responsible for coronavirus disease 2019 (COVID-19), and is the seventh member of the human coronavirus family, causing symptoms such as cough and fever, severe pneumonia and death, assuming pandemic characteristics [1,2].

Approximately 5–20% of patients with SARS-Cov-2 pneumonia are admitted to intensive care units (ICUs), with a mortality rate between 26 and 61.5%. Most patients present respiratory failure and over 88% require invasive mechanical ventilation (IMV) [3,4]. Age, body mass index (BMI) > 25 kg/m^2^, as well as a history of oncological, cerebrovascular, chronic kidney disease, chronic obstructive pulmonary disease, diabetes mellitus (DM), heart diseases (cardiomyopathies, heart failure, coronary heart disease), hypertension, and smoking (past or present), among others, have been identified as risk factors associated with the development of severe disease [5].

Ventilation in the prone position (PP) has been used for decades in acute respiratory distress syndrome (ARDS) and is recommended by the main international health organizations as an alveolar recruitment maneuver to improve oxygenation [6], and is considered a safe strategy associated with a reduced mortality rate in patients with moderate to severe ARDS [6,7,8,9], according to the current Berlin definition [10].

Based on the pathophysiology and results of PP in patients with ARDS, its application was extended to patients with SARS-Cov-2 pneumonia and is recommended by the World Health Organization (WHO) and the Surviving Sepsis Campaign for a period of 12–16 h in patients under IMV with COVID-19 and moderate to severe ARDS [11,12]. In Portugal, the Sociedade Portuguesa de Cuidados Intensivos (SPCI) and the Infeção e Sépsis Group issued recommendations for the approach to COVID-19 in intensive care medicine, advising early PP if arterial oxygen pressure/inspired oxygen fraction (PaO_2_/FiO_2_) ratio <150 mmHg, for minimum periods of 16 h and with use of neuromuscular blockers (NMB) for ≤48 h if frequent dyssynchrony [13]. The pressure exerted by the pandemic has led health systems worldwide to develop strategies to manage available resources. In this context, studies have emerged recommending the extension of the duration of PP up to 36 h, arguing that there is an improvement in the PaO_2_/FiO_2_ ratio higher compared to conventional PP (16 h), remaining significantly higher in the return to dorsal decubitus (DD), with a reduction in the number of PP cycles performed [3,14]. The need to optimize resources led to the extension of the applicability of PP to awake patients under supplemental oxygen, high-flow oxygen therapy (HFO), and non-invasive ventilation (NIV), with improved oxygenation and with the advantage that it can be performed in an inpatient setting [15,16]. However, the literature is not consensual and there are studies showing that the response of these patients to PP is very varied and that the improvement in oxygenation obtained does not translate into clinical improvement or favorable radiological evolution, suggesting that PP does not offer more benefits than a transitory improvement in oxygenation [17,18]. Regarding the use of PP in conscious patients, the literature is equally controversial and there is no evidence that PP in these patients reduces the need for orotracheal intubation (OTI) and in some cases, there is a delay in OTI and increase in emergency OTI [3,19].

Based on these considerations and to improve the approach to patients with SARS-Cov-2 pneumonia in future situations, a research study was carried out at the Hospitalar Centre of Tâmega and Sousa (CHTS) Multipurpose Intensive Care Unit (MICU) with the aim of assessing the effects of PP on the outcomes of patients with SARS-Cov-2 pneumonia.

## 2 Methods

### 2.1 Type of study

This is a quantitative approach, quasi-experimental single-group, longitudinal and retrospective study.

### 2.2 Population and sample

The sampling method selected was non-probabilistic sampling by rational choice, with the individuals being selected according to a set of pre-defined inclusion criteria in order to represent the phenomenon under study, with the main limitation of this technique being less reliable than probabilistic methods when it comes to generalizing the results to our population [20].

Patients admitted to the MICU under study, diagnosed with SARS-Cov-2 pneumonia, and submitted to IMV and PP between March 23 and December 27, 2020 were included in the study. The application of the inclusion and exclusion criteria resulted in a sample of 52 patients.

The research followed all ethical principles, in accordance with the Helsinki standards, and the research was approved by the Ethics Committee of the CHTS.

### 2.3 Instrument for data collection

Data collection was carried out by consulting clinical files.

### 2.4 Operationalization and categorization of variables

The variables under study were divided into eight groups, namely:

1. Demographic data encompassing the variables: gender and age.
2. Clinical data, where the variables are inserted: BMI, diagnostic category, relevant antecedents, provenance and nursing manpower use (NMU).
3. Disease chronology, which covers the following variables: onset of symptoms, timing to MICU and timing to OTI.
4. Adjuvant therapies encompass the use of NMB, amines, tracheostomies and extracorporeal membrane oxygenation (ECMO).
5. PP characteristics include the variables: timing to first PP, number of PP cycles per patient and PP time.

Gasometric data including oxygenation translated by the PaO_2_/FiO_2_ ratio were assessed at three different moments: before PP (T0), during PP (T1) and after PP (T2 (at the return to the DD), by means of control gasometry collected 1–3 h before, during and after PP.

The response to PP was classified as follows: no response, poor response, good response and very good response, according to the evolution of the PaO_2_/FiO_2_ ratio before and after PP; patients who presented a mean PaO_2_/FiO_2_ ratio after PP lower than before PP were considered no responders to PP. In order to classify the response to PP, the mean percentage of variation between the PaO_2_/FiO_2_ ratio before and after PP was assessed; a variation below the mean was considered a poor response to PP [19]; a variation between the mean and twice the mean was considered a good response and a very good response when the variation in the PaO_2_/FiO_2_ ratio before and after PP was more than twice the mean variation.

### 2.5 Data analysis

For data processing, a database was built in the Statistical Program for Data Processing in Social Sciences (SPSS, version 26.0), in which they were edited, considering a type I error probability (*α*) of 0.05. Data were analyzed using descriptive statistics by calculating the absolute and relative frequencies for all variables and the measures of central tendency and dispersion for the ratio measurement level variables. In addition, inferential statistics was performed using the ANOVA test of repeated measurements to assess the significance of the effectiveness of the positioning in PP on the PaO_2_/FiO_2_ ratio and the evolution of this ratio in the three moments under analysis. Multivariate analysis using an ordinal regression model was also used to test the influence of independent variables (demographic variables, relevant clinical history, disease chronology and PP characteristics) on the dependent variable “response to PP.” Then, the Kruskal–Wallis test was used to compare the means of ordination of the categories of the dependent variables (duration of IMV and length of stay in the MICU) according to the response to PP. Finally, we used the *χ* ^2^ test to check for statistical differences between the response to PP regarding the incidence of tracheostomy and mortality rate.

## 3 Results

From the total sample (*n* = 52 patients), there was a predominance of males (57.7%), with a mean age of 63.9 ± 10.2 years (Δ42–83). Most patients (69.2%) had relevant clinical history, the most frequent being hypertension (HT) (77.8%), DM (47.2%) and obesity (38.5%); the mean BMI was 28.86 ± 5.27 (Δ20–44.4). The mean NMU of these patients was 31.54 ± 3.61.

The onset of symptoms appeared on average 6.6 ± 3.9 days before hospital admission, and they were transferred to the MICU at 2.5 ± 3(2) days of admission.

In the MICU, OTI was performed within the first 15.3 ± 30.3(1) h after admission. Patients remained ventilated for a mean period of 13 ± 9.7 days, with a mean stay in the MICU of 15.9 ± 11 days. Almost all patients (98.1%) required NMB, 98.1% required aminergic support, 5.8% were transferred to other hospital units and submitted to extracorporeal oxygenation membrane (ECMO) and 17.3% were tracheostomized. The mortality rate was 50%.

The first PP cycle occurred, on average, 45.7 ± 61.2(21) h after OTI. Each patient performed an average of 2.8 ± 1.6 cycles, lasting 20.5 ± 3.7 h.

The mean PaO_2_/FiO_2_ ratio before starting PP was 115.6 ± 38.2 in PP of 159.1 ± 65.4 and after undoing PP of 140.1 ± 48.9. It was found that 23.1% of the patients did not respond to PP, 26.9% had a poor response, 23.1% had a good response and 26.9% had a very good response. The mean variation between PaO_2_/FiO_2_ before and after PP was 21.27%.

The inferential analysis showed that oxygenation differs significantly between the three moments of assessment of patients undergoing PP, observing significant differences between the mean PaO_2_/FiO_2_ ratios before, during and after PP until the third cycle of PP (Table 1).

**Table 1:** Repeated measures ANOVA test to compare the mean of the PaO_2_/FiO_2_ ratio at three assessment times, Penafiel, Portugal, 2020 (*N* = 52)

Dependent variables	Cycle *N* PP	Mean ± dp	Z	*p*	Eta partial square	Power observed
P/F (*T*0)	1	112.59 ± 38.24	21.198	0.000	0.306	1.000
P/F (*T*1)		168.06 ± 69.9				
P/F (*T*2)		156.98 ± 46.96				
P/F (*T*0)	2	130.85 ± 34.31	8.46	0.001	0.204	0.959
P/F (*T*1)		168.50 ± 64.93				
P/F (*T*2)		145.79 ± 48.94				
P/F (*T*0)	3	117.35 ± 27.05	13.831	0.000	0.386	0.997
P/F (*T*1)		167.17 ± 59.55				
P/F (*T*2)		135 ± 38.50				
P/F (*T*0)	4	117.71 ± 34.39	3.827	0.35	0.227	0.643
P/F (*T*1)		141.5 ± 43.58				
P/F (*T*2)		120.29 ± 54.61				
P/F (*T*0)	5	84.67 ± 46.1	0.336	0.72	0.46	0.093
P/F (*T*1)		100.5 ± 42.5				
P/F (*T*2)		102.87 ± 50.71				
P/F (*T*0)	6	78 ± 15.38	5.7	0.06	0.66	0.640
P/F (*T*1)		122.25 ± 42.74				
P/F (*T*2)		86 ± 21.21				

Cycle *N* PP – number of cycles of PP; dp – standard deviation; *Z*-test value; *p* – likelihood; Eta partial square – effect size; *T*0 – before PP, *T*1 – during PP, *T*2 – after PP (on return to DD).

Regarding the effects of PP characteristics and disease chronology on the response to PP, an adjusted ordinal model was found to be equal to −0.456 for the number of PP cycles, and −0.020 for the timing to OTI, i.e. the response to PP is inversely proportional to the number of PP cycles and the timing to OTI. For the remaining variables of disease chronology and PP characteristics, there was no ordinal adjusted model (*p* > 0.05). According to the model, as the timing to OTI and the number of cycles of PP increase, the probability values of a higher response to PP are 0.036 and 0.009, respectively (Table 2).

**Table 2:** Ordinal regression model for parameter estimation relevant to demographic variables and clinical background under the response to PP, Penafiel, Portugal, 2020 (*N* = 52)

Variables	Estimate	Standard deviation	*X* ^2^ Wald	df	*p*	Confidence interval 95%
No reply to PP	4.227	3.981	1.127	1	0.288	[−3.576; 12.031]
Poor response to PP	5.687	4.026	1.996	1	0.158	[−2.203; 13.577]
Good response to PP	6.943	4.059	2.926	1	0.087	[−1.012; 14.898]
No smoking	2.219	1.028	4.660	1	0.031	[0.204; 4.234]

df – degrees of freedom; *p* – probability.

The model is statistically significant (*χ* ^2^ = 13.914; *p* = 0.031), and the effect size is small (McFadden = 0.097; NagelKerke = 0.251; Cox and Snell = 0.235). Regarding the influence of demographic variables and relevant clinical history on the response to PP, an ordinal adjusted model (= +2.219, no Smoking) was found. For the remaining demographic variables and relevant clinical history, an ordinal adjusted model was not found (*p* > 0.05). According to the model, as the proportion of patients with no history of smoking increases, the probability of observing a higher response to PP is 0.031 (Table 3).

**Table 3:** Ordinal regression model for estimates of the parameter’s chronology of illness and PP characteristics under the response to PP, Penafiel, Portugal, 2020 (*N* = 52)

Variables	Estimate	Standard deviation	X^2^ Wald	df	*p*	Confidence interval 95%
No reply to PP	−0.324	1.634	0.039	1	0.843	[−3.526; 2.879]
Poor response to PP	1.105	1.636	0.456	1	0.500	[−2.102; 4.311]
Good response to PP	2.293	1.663	1.901	1	0.168	[−0.966; 5.551]
Timing to EOT	−0.020	0.009	4.379	1	0.036	[−0.038; −0.001]
No. of PP cycles	−0.456	0.175	6.750	1	0.009	[−0.800; −0.112]

df – degrees of freedom; *p* – probability.

However, the model is not statistically significant (*χ* ^2^ = 16.340; *p* = 0.219) and the effect size is small (McFadden = 0.114; NagelKerke = 0.288; Cox and Snell = 0.270).

Finally, regarding the outcome of patients, we found that the mean scores for the duration of IMV and length of stay in the MICU of patients with different responses to PP did not differ significantly (*p* = 0.123 and 0.263) (Table 4).

**Table 4:** Kruskal–Wallis test to compare the mean rank of IMV time and length of stay of patients in the MICU according to the response to PP, Penafiel, Portugal, 2020 (*N* = 52)

Dependent variable	Response to PP	*N*	Mean rank	Test value	df	*p*
IMV time	No	12	22.33	KW = 5.782	3	0.123
	Weak	14	30.07			
	Good	12	32.17			
	Very good	14	21.64			
Length of stay in MICU	No	12	21.58	KW = 3.989	3	0.263
	Weak	14	29.57			
	Good	12	30.38			
	Very good	14	24.32			

KW – Kruskal–Wallis.

There were also no statistically significant differences regarding the incidence of tracheostomy and mortality (*χ* ^2^: *p* ≥ 0.172; 0.148) (Table 5).

**Table 5:** *χ* ^2^ test for differences between the proportions of the categories of nominal dependent variables of hypothesis H4 as a function of response to PP, Penafiel, Portugal, 2020 (*N* = 52)

Dependent variables	Response to PP				*n*	Test value	df	*p*
	No	Weak	Good	Very good				
**Tracheostomy**								
No AR	11 (91.7%)	9 (64.3%)	10 (83.3%)	13 (92.9%)	43	*χ* ^2^ = 5.004	3	0.172^a^
	0.9	−2.1	−0.1	−1.2				
Yes AR	1 (8.3%)	5 (35.7%)	2 (16.7%)	1 (7.1%)	9			
	−0.9	2.1	−0.1	−1.2				
**Result**								
Deceased AR	9 (75%)	8 (57.1%)	6 (50%)	3 (21.4%)	26	9.482	6	0.148ª
	2	0.6	0	−2.5				
Hospital transfer AR	1 (8.3%)	0 (0.0%)	1 (8.3%)	1 (8.3%)	3			
	0.4	−1.1	0.4	0.3				
Improved AR	2 (16.7%)	6 (42.9%)	5 (41.7%)	10 (71.4%)	23			
	−2.2	−0.1	−0.2	2.4				

*n* – absolute frequency; df – degrees of freedom; *p* – probability; AR – adjusted residual; ^a^ – Pearson’s Chi-square test.

It should be noted that PP was performed by maintaining hemodynamic stability, and there was only a slight increase in the mean arterial pressure (MAP) and heart rate (HR) after PP. A total of 143 PP were performed, with complications arising in only 16.2% of the PP performed, which mainly occurred in the first and second PP cycles, the most frequent being edema of the face/eyes/lips (39.1%), followed by pressure ulcers (PU) (30.4%).

## 4 Discussion

The profile of patients who developed a severe disease and underwent PP is in line with that described in the literature, being characterized by elderly male patients, with a clinical history of HT, DM and obesity, with a high nursing workload index [2,12,15,16,19]. With regard to gender, although the current knowledge is still very limited, some studies point out that male patients with SARS-cov-2 pneumonia tend to have a higher mortality rate [21,22]. However, the response to PP does not seem to be influenced by gender. However, in conscious patients who underwent PP, it was considered a favorable procedure predominantly by females (73% females versus 56.5% males), which seems to be related to differences in literacy and educational attainment [22].

The PP was performed according to recommendations [4,9,10,11,12] at the time in force, with the first PP cycle occurring 45.7 ± 61.2(21) h after OTI, with each patient performing an average of 2.8 ± 1.6 cycles lasting 20.5 ± 3.7 h. The mean PaO_2_/FiO_2_ ratio before starting PP was 115.6 ± 38.2; at PP, the ratio was 159.1 ± 65.4 and after undoing PP, the ratio was 140.1 ± 48.9, showing an improvement of oxygenation with PP. The mean increase between PaO_2_/FiO_2_ before and after PP was 21.27%. In other studies on the subject, PP was also associated with improvement in oxygenation [6,14,23,24], with a mean variation between PaO_2_/FiO_2_ before and after PP higher than that observed in this study (49%) [21], maintaining the improvement of oxygenation on return to the DD, starting PP earlier, with a mean PaO_2_/FiO_2_ ratio before starting the PP of 150 (IQR, 125–183), in PP of 232 (IQR, 174–304) and after undoing PP of 217 (IQR, 149–263), performing each patient 2 (3–5) sessions of PP with an average of 18 h (IQR, 16–22 h) per session[4], 3 cycles been necessary with about 36 h in other studies [3].

As expected and observed in other studies, PP was found to improve the PaO_2_/FiO_2_ ratio in patients with SARS-Cov-2 pneumonia [4,6,14,21,24,25]. However, significant differences between mean PaO_2_/FiO_2_ ratios before, during and after PP are only observed up to the third PP cycle. From the fourth cycle onwards, the PaO_2_/FiO_2_ ratio (T0) starts decreasing and PP is not sufficient to reverse hypoxia. It was found that the response to PP is inversely proportional to the number of PP cycles and OTI timing. In other studies, it was found that by prolonging the stay in PP up to 36 h, a reduction in the number of cycles of PP was achieved, which translated into an improvement in the PaO_2_/FiO_2_ ratio, which was perpetuated upon the return to DD [23]. Other authors found, through a systematic review of literature, that PP improved oxygenation in patients under IMV, from the second cycle and in prolonged episodes of PP [26] but no study refers to the decrease in the efficacy of PP with the increase in the number of cycles, nor to the underlying mechanisms.

Despite the high incidence of relevant clinical history and demographic differences observed as a function of response to PP, contrary to expectations, it was found that the response to PP is not influenced by these variables. On the other hand, it was observed that response to PP did not influence the patient outcome, with no significant differences in IMV time, length of stay, incidence of tracheostomy and mortality. Although these results differ from what was expected for this study, other researchers also observed that 80% of patients had improved oxygenation with PP, although there was no correlation between this improvement and clinical improvement [4], with only a trend toward a non-significant reduction in mortality (OR: 0.76; 95% CI: 0.54–1.06; *p* = 0.11, I2 63%) [8].

It should be noted that PP was performed without hemodynamic repercussions, with complications occurring in only 16.2% of the PP performed, suggesting that it is safe to be performed.

This study characterized patients with SARS-Cov-2 pneumonia, described the use of PP as an alveolar recruitment maneuver in these situations and characterized the response to PP based on its effects on oxygenation to understand the influence of demographic variables, relevant clinical history and disease chronology, as well to analyze their impact on patient outcomes.

The results observed have implications for the care of critically ill patients with SARS-Cov-2 pneumonia because, in addition to having demonstrated that PP is a safe procedure that improves oxygenation in these patients, it also showed that multiple PP sessions are not beneficial. Alternative treatments should be considered for patients requiring more than three cycles of PP, and it is essential to avoid delaying OTI in order to improve the response to PP.
